# Effectiveness of endoscopic totally extraperitoneal (TEP) hernia correction for clinically occult inguinal hernia (EFFECT): study protocol for a randomized controlled trial

**DOI:** 10.1186/s13063-018-2711-7

**Published:** 2018-06-18

**Authors:** Marleen M. Roos, Egbert-Jan M. M. Verleisdonk, Floris B. M. Sanders, Arno W. Hoes, Rebecca K. Stellato, Geert W. J. Frederix, Rogier K. J. Simmermacher, Josephina P. J. Burgmans, EFFECT study group, Marleen M. Roos, Coen V. van Hessen, Wouter J. Bakker, Egbert Jan M. M. Verleisdonk, Floris B. M. Sanders, Ine P. J. Burgmans, Rogier K. J. Simmermacher, Arno W. Hoes, Rebecca K. Stellato, Geert W. Frederix, Henk F. van Stel, Andre de Vries, Maarten P. Simons, Paul M. Verheijen, Caroline M. E. Contant, Johannes A. Wegdam, Gerrit D. Slooter, Huib A. Cense, Frank W. H. Kloppenberg

**Affiliations:** 1 Department of Surgery/Hernia Clinic, Diakonessenhuis, Utrecht/Zeist, The Netherlands; 2 Department of Radiology, Diakonessenhuis, Utrecht/Zeist, The Netherlands; 3 Julius Center for Health Sciences and Primary Care, University Medical Centre Utrecht, Utrecht, The Netherlands; 4 Department of Surgery, University Medical Centre Utrecht, Utrecht, The Netherlands

**Keywords:** Inguinal hernia, Clinically occult, TEP, Watchful waiting, Pain, Quality of life, Cost-effectiveness

## Abstract

**Background:**

Groin pain is a frequent complaint in surgical practice with an inguinal hernia being at the top of the differential diagnosis. The majority of inguinal hernias can be diagnosed clinically. However, patients with groin pain without signs of an inguinal hernia on anamnesis or physical examination provide a diagnostic challenge. If ultrasonography shows a hernia that could not be detected clinically, this entity is called a clinically occult hernia. It is debatable if this radiological hernia is the cause of complaints in all patients with inguinal pain.

The objective of this study is to assess whether watchful waiting is non-inferior to endoscopic totally extraperitoneal (TEP) inguinal repair in patients with a clinically occult inguinal hernia.

**Methods:**

The EFFECT study is a multicenter non-blinded randomized controlled non-inferiority trial. Adult patients with unilateral groin pain and a clinically occult inguinal hernia are eligible to participate in this study. A total of 160 participants will be included and randomized to TEP inguinal hernia repair or a watchful waiting approach. The primary outcome of this study is pain reduction 3 months after treatment, measured by the Numeric Rating Scale (NRS). Secondary outcomes are quality of life, cost-effectiveness, patient satisfaction and crossover rate. Eight surgical centers will take part in the study. Participants will be followed-up for 1 year.

**Discussion:**

This is the first large randomized controlled trial comparing treatments for patients with groin pain and a clinically occult inguinal hernia. To date, there are no interventional studies on the effect of surgery or a watchful waiting approach in terms of pain or quality of life in this subset of patients. A trial comparing the outcomes of the two approaches in patients with a clinically occult inguinal hernia is urgently needed to provide data facilitating the choice between the two treatment options. If watchful waiting is not inferior to surgical repair, costs of surgical repair may be saved.

**Trial registration:**

The study protocol (NL61730.100.17) is approved by the Medical Ethics Committee (MEC-U) of the Diakonessenhuis, Utrecht, The Netherlands. The study was registered at the Netherlands Trial Registry (NTR6835) registered on November 13, 2017.

**Electronic supplementary material:**

The online version of this article (10.1186/s13063-018-2711-7) contains supplementary material, which is available to authorized users.

## Background

Groin pain is a frequent complaint in surgical practice that encompasses a large number of possible etiologies. A well-known cause of groin pain is an inguinal hernia. Elective inguinal hernia correction is the most commonly performed operation worldwide with an estimated 30.000 procedures in the Netherlands annually [1].

In the majority of cases, an inguinal hernia can be diagnosed clinically. A classical hernia presents as a reducible groin swelling with a positive cough impulse (Valsalva manoeuvre), with or without the presence of discomfort. However, patients with groin pain without signs of an inguinal hernia on anamnesis or physical examination provide a diagnostic challenge.

For patients with groin pain in whom no swelling can be identified, current guidelines advise ultrasonography of the groin, followed by MRI if ultrasonography is inconclusive [2, 3]. When additional imaging shows a hernia that could not be detected clinically, this entity is called a clinically occult hernia. The current guidelines do not provide a specific therapeutic approach for this type of hernias, and for unclear reasons the radiologic presence of a clinically occult inguinal hernia often leads to surgical intervention [2, 3].

However, it is debatable if in all cases a symptomatic inguinal hernia truly exists or whether the hernia on additional imaging is an incidental finding with an alternative cause for the pain complaints. The radiologic finding might even be false-positive in some cases. Considering this, it is likely that not in all patients with a clinically occult hernia a surgical procedure is justified, and it is possible that (chronic) pain complaints persist or increase after surgery. This consideration is important for adequate patient information and avoidance of unnecessary surgical interventions.

The incidence of groin pain in combination with a clinically occult inguinal hernia is not well described in literature. A prospectively registered database kept in the Hernia Clinic of the Diakonessenhuis (Diakonessenhuis Utrecht/Zeist, The Netherlands) shows inguinal pain without symptoms of an inguinal hernia on physical examination or anamnesis but presence of an inguinal hernia on ultrasonography in 9.5% of patients.

Up till now, there are no studies performed specifically focusing on the course of pain and quality of life after correction of a (clinically) occult inguinal hernia or a watchful waiting approach. A trial comparing the outcomes of the two approaches in patients with a clinically occult inguinal hernia is urgently needed.

## Methods

### Objective

The objective of this study is to assess if watchful waiting is non-inferior to endoscopic totally extraperitoneal (TEP) inguinal repair in patients with a clinically occult inguinal hernia.

### Study design

The EFFECT trial is designed as a non-blinded, randomized controlled non-inferiority trial comparing surgical treatment by TEP repair to watchful waiting in patients with a clinically occult inguinal hernia. This study is conducted in accordance with the principles of the Declaration of Helsinki and Good Clinical Practice guidelines. The independent ethics committee of the Diakonessenhuis Utrecht (MEC-U) has approved this study protocol (protocol number NL61730.100.17). Eight Dutch centers participate in this study: Diakonessenhuis Utrecht/Zeist, Meander Medical Center Amersfoort, Onze Lieve Vrouwe Gasthuis (OLVG) Amsterdam, Maasstad Hospital Rotterdam, Maxima Medical Center (MMC) Veldhoven, Elkerliek Hospital Helmond, Rode Kruis Hospital (RKZ) Beverwijk and Treant Care Group Hoogeveen.

Patient recruitment started on December 29, 2017. Written informed consent will be obtained from all participants by the study coordinator. Inclusion will take up to a maximum of 2.5 years and participants will be followed up for 1 year in total. The total duration of the study will be 3.5 years. All study participants will be asked to fill out written questionnaires at different time points (baseline, 1.5, 3, 6 and 12 months following treatment) and will be scheduled for physical examination by experienced hernia surgeons at two additional time points at the surgical outpatient clinic for the purpose of this study Participants can withdraw from the study at any time, for any reason.

Randomization, collection and storage of study data will take place through a uniform electronic case report form (eCRF).

**Fig. 1:**
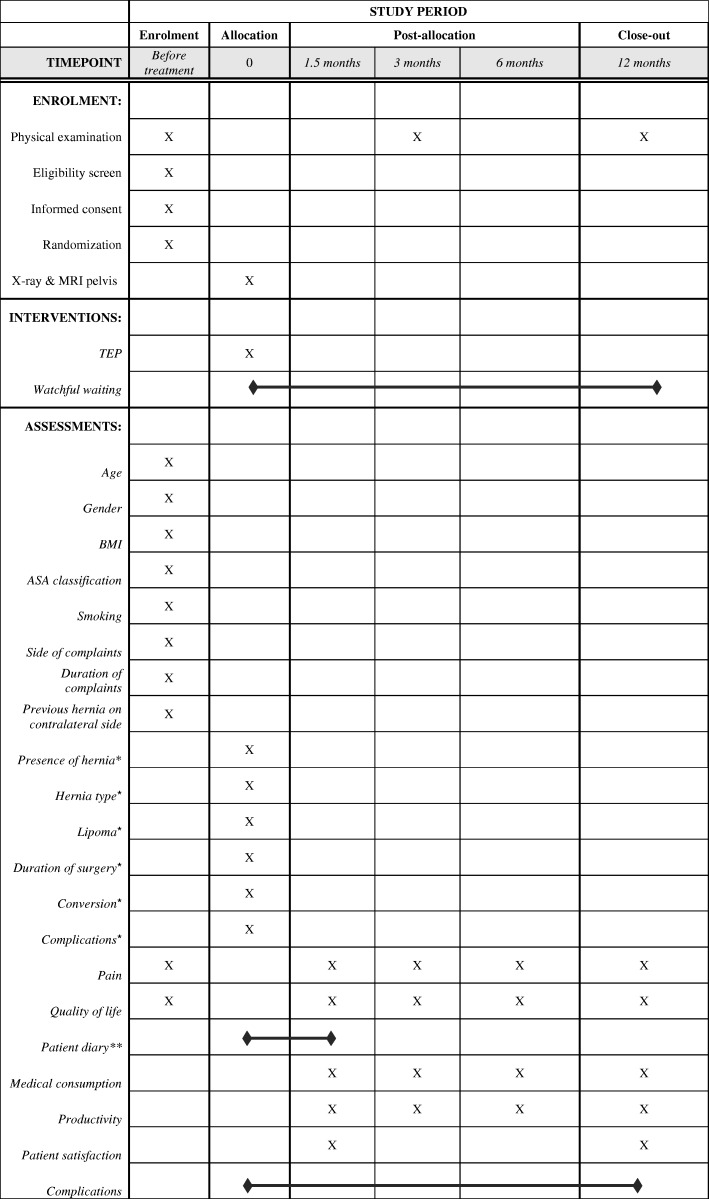
Content for the schedule of enrolment, interventions and assessments according to the SPIRIT statement. *ASA* American Society of Anesthesiologists, *BMI* body mass index, *MRI* magnetic resonance imaging, *TEP* totally extraperitoneal, *Findings upon operation in case of randomization to totally extraperitoneal (TEP) hernia repair. **Patient diary regarding physiotherapy and use of painkillers

A SPIRIT figure and checklist for this study protocol are provided in Fig. 1 and Additional file 1, respectively.

### Participants

Adult patients with groin pain and a clinically occult inguinal hernia are eligible to participate in this study*.*

**Fig. 2:**
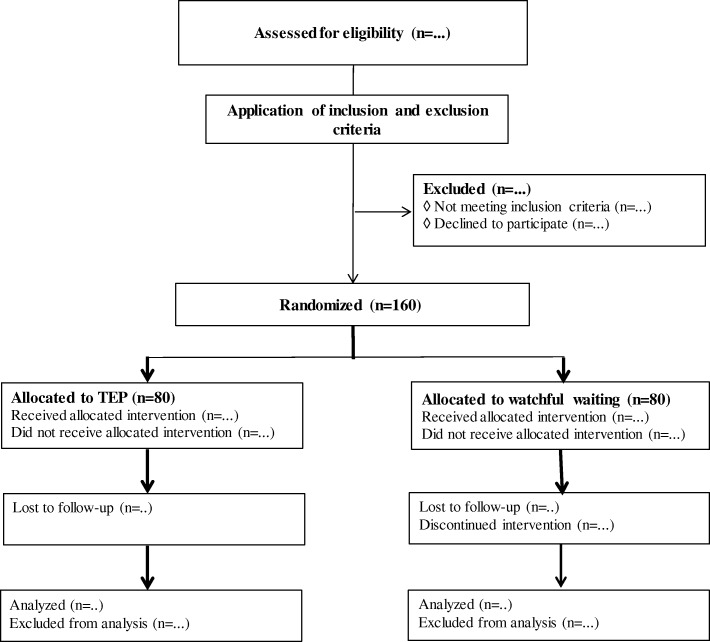
Study flow chart. *TEP* totally extraperitoneal

All patients presenting to the outpatient clinics of participating centers are physically examined by experienced hernia surgeons and screened for trial eligibility (Fig. 2). Patients will be informed and included at the surgical outpatient department at one of the participating centers.

Each subject must meet the following inclusion criteria:

- Age ≥ 18 years
- Unilateral groin pain
- No features of an inguinal hernia on anamnesis (no visible or palpable groin swelling)
- No features of an inguinal hernia on physical examination (no visible or palpable groin swelling and a negative Valsalva manoeuvre)
- Radiological diagnosis of an inguinal hernia on ultrasonography

A potential subject who meets any of the following criteria will be excluded from participation:

- Previous inguinal hernia on the symptomatic side
- Previous surgery in inguinal region of the symptomatic side
- Body mass index (BMI) ≥ 40
- American Society of Anesthesiologists (ASA) classification > III
- Factors that complicate follow-up by means of questionnaires (e.g. language barrier, psychiatric disorders)
- Unwillingness to undergo surgery

### Randomization

After informed consent is obtained, the study coordinator will directly randomize patients to either TEP repair or a watchful waiting approach by means of an online random treatment generator, stratified by center.

The surgeon, patient and coordinating researcher are not blinded for the allocated treatment.

### Baseline assessment

At baseline, all participants are asked to fill out electronic questionnaires. Also, all participants will undergo X-ray and magnetic resonance imaging (MRI) of the pelvis, to be able to assess baseline comparability of the groups in a later stage. The surgeon will be blinded to the outcomes of these investigations and they do not influence treatment. Only in rare severe cases (e.g. a tumor or fracture) radiologists are instructed to contact the treating physician.

### Intervention

Patients will be randomized to TEP inguinal hernia repair or a watchful waiting approach.

#### TEP inguinal hernia repair

The patients randomized to an operative treatment will undergo endoscopic totally extraperitoneal (TEP) inguinal hernia repair. This procedure will be standardized according to current guidelines in all participating centers [2, 3].

A preperitoneal synthetic mesh will be placed in a standardized manner. Perioperative findings (presence of an inguinal hernia, classification of inguinal hernia according to the European Hernia Society (EHS) hernia classification, presence of a lipoma) and perioperative complications are recorded in the operation chart [2, 3].

#### Operative procedure

After induction of general anaesthesia, a sub-umbilical incision is made. The anterior rectus sheath is divided transversely to expose the rectus muscle, which is retracted laterally. A 10 mm trocart is inserted into the preperitoneal space after which the preperitoneal space is created digitally (or with a balloon) and insufflated. A 5 mm trocart is placed at midline between umbilicus and symphysis, after which the pubic bone and cavum Retzii are dissected. Below the level of the epigastric vessels lateral dissection takes place, and a second 5 mm trocart is placed laterally. (Another option is placement of the second 5 mm trocart in the midline, below the first 5 mm trocart). A possible lateral hernia sac is dissected with identification of the vas deferens and vessels. A possible medial of femoral hernia is reduced. The peritoneum is dissected cranially. Possible lipomas are identified and reduced or resected. A synthetic mesh is introduced and positioned against the anterior abdominal wall, covering the internal ring, the femoral canal and the medial space. Next, under endoscopic sight the preperitoneal space is desufflated, and all trocarts are removed. The rectus sheath is closed with vicryl, the dermis is closed with monocryl.

#### Postoperative care

Patients are discharged at the day of surgery, unless complications prohibit early discharge.

Participants are advised to avoid strenuous physical activity during the first postoperative week.

#### Surgical quality control

A selected number of trained surgeons will perform TEP hernia repair. Hereby, we maintain quality of the operations and minimize differences in success rates. Surgeons in the participating centers have completed their learning curve and are sufficiently experienced (> 250 procedures per individual surgeon) in TEP inguinal hernia repair.

#### Watchful waiting

Patients in the watchful waiting study arm will be treated with rest, pain killers and optional physical- and/or physiotherapy. It is expected that the patients in this group will present with a variety of complaints and will not experience the same (intensity of) complaints, therefore we cannot standardize the treatment in this study arm. Intensity and frequency of this treatment will depend on the amount of complaints the individual patient is experiencing and is expected to differ between patients. The decision to offer physical- and/or physiotherapy will be in the hands of the treating physician, who will decide if he or she suspects the kind of complaints where physical- and/or physiotherapy will be of help.

### Outcomes

The primary outcome will be pain reduction after 3 months, measured at rest and during physical activity. The first pain score is obtained at baseline. The first three questions of the validated EuraHS Quality of Life (EuraHS-QoL) reflect intensity of pain on an 11-point numeric rating scale (NRS) (0–10 where 0 reflects “no pain” and 10 reflects “the worst possible pain”) [4]. Patients rank the intensity of their pain on this scale at rest (specified as lying down) and during physical activity (defined as walking, cycling or practicing sports). The third question is the amount of pain felt during the last week, so insight is also provided in the most recent pain the patient experienced. Follow-up pain levels are determined at three additional time points (1.5, 6 and 12 months following treatment) as secondary outcome parameters.

Secondary outcomes include quality of life, cost-effectiveness, patient satisfaction and crossover rate. Quality of life will be assessed by the validated EuraHS-QoL and Euro Quality of Life-5D-5 L (EQ5D-5 L) questionnaires at baseline, and 1.5, 3, 6 and 12 months postoperatively [5].

Cost-effectiveness will be based on all resources used within and outside the hospital and productivity loss for both groups of patients. The acquired data will consist of all health care professional visits, hospitalizations, imaging, biochemical investigations and surgery. Health care use will be monitored through the medical consumption questionnaire (MCQ), a generic instrument for measuring health care use and calculating medical costs, at 1.5,3, 6 and 12 months after treatment [6]. Participants in both treatment groups will be given a patient diary where the use of painkillers and (optional) treatment with physical- and/or physiotherapy will be registered the first 6 weeks after treatment. Productivity will be measured through the productivity cost questionnaire (PCQ), a standardized instrument for measuring and valuing productivity losses, at 1.5,3,6 and 12 months after treatment [7]. We aim to calculate total costs per patient in every group. We aim to calculate cost-effectiveness ratios to indicate the total costs per additional unit of effect, cost per quality-adjusted life year (QALY).

Patient satisfaction will be measured at 3 and 12 months after treatment. The measuring instrument for the outcome parameter patient satisfaction is a self-designed 11-point scale ranging from 0 (“no satisfaction”) to 10 (“total satisfaction”).

The crossover rate will reflect the percentage of patients initially assigned to the watchful waiting group that crosses over to surgical treatment.

Other study parameters include baseline characteristics (gender, age, ASA classification, BMI, smoking, medication, side of complaints, duration of complaints, previous inguinal hernia on contralateral side), perioperative outcomes (presence of a hernia, hernia type, presence of lipoma, duration of surgery, conversion, complications) and postoperative complications. Also, the percentage of clinically occult hernias in the watchful waiting group that develop into clinically overt inguinal hernias will be assessed.

### Sample size calculation

The sample size determination is based on the assumption that a watchful waiting approach is non-inferior to a TEP inguinal hernia correction; from this assumption there is no expected difference between the difference scores of both groups. No consensus exists concerning a minimal clinically relevant difference on the NRS, though previously published literature describes a difference of one point [8]. We used three-quarters of a point (0.75) on the NRS scale as an equivalence margin, using the NRS as a continuous variable. Expected variance in the primary outcome was estimated from a prospectively collected database containing 919 patients at the Diakonessenhuis; a standard deviation of 2.3 was found for the difference scores, and a correlation of 0.8 was found between pretreatment pain and change in pain scores. To be able to detect an equivalence margin of 0.75 on change on the NRS scale between the groups, with a power of 90% and a one-sided alpha of 0.025, a sample size of 199 patients in each arm is required. Because we will correct for baseline pain intensity, an analysis of covariance (ANCOVA) correction was used, resulting in 72 patients per arm [9]. Taking a loss-to-follow-up of 10% into account, the total sample size should contain at least 160 patients with 80 patients per arm.

### Statistical analysis

#### Primary outcome measure

The primary outcome measure is the difference in pain intensity (NRS during rest/physical activity) 3 months following treatment compared to baseline. Difference scores of the NRS (posttest minus pretest) will be used in both treatment groups. The NRS score will be used as a continuous variable. For comparison of difference scores an ANCOVA will be used, in which the estimated difference in pain scores will be corrected for the pain intensity at baseline.

#### Secondary/other study parameter(s)

Continuous secondary outcomes 3 months following treatment will be analyzed similarly to the primary outcome, with an ANCOVA on the difference scores correcting for baseline scores. Outcome measures at 1.5,3,6 and 12 months following treatment will be analyzed using linear mixed-effects models to adjust for repeated measurements within individuals. Contrasts will be used to investigate differences between the study arms at the post-treatment time points.

The primary analysis of outcome parameters is an intention-to-treat analysis. Primary and secondary outcomes 3 months after treatment will also be evaluated only for the patients who remained in the arm to which they were randomized (per protocol analysis).

Data will be analyzed by means of IBM SPSS Statistics version 23 or higher (IBM Corp, Armonk, NY, USA).

## Discussion

This is the first randomized controlled trial comparing surgery to a watchful waiting approach in the specific subset of patients with a clinically occult inguinal hernia.

The current guidelines provide no clear advice on the treatment of the particular subset of patients that has a clinically occult inguinal hernia [2, 3]. To date, we could find no intervention studies on the effect of surgery or a watchful waiting approach in terms of pain or quality of life in patients with a clinically occult inguinal hernia. Up till now, all the studies performed on clinically occult inguinal hernias were diagnostic and aimed to determine the diagnostic value for the diagnosis of occult hernia of imaging modalities in patients with groin pain and no findings of an inguinal hernia on physical examination [10–22].

A trial comparing the outcomes of the two approaches in patients with a clinically occult inguinal hernia is urgently needed to provide data facilitating the choice between the two treatment options. With this trial, we aim to determine which treatment strategy is the most (cost-)effective for this particular subset of patients. If watchful waiting is non-inferior to surgical repair, costs of surgical repair and complications may be saved.

### Trial status

The study was opened to recruitment in December 2017. Recruitment is ongoing. The duration of the study period will be 3.5 years.

## Additional file

Additional file 1: SPIRIT checklist for recommended items to address in a clinical trial protocol and related documents. (PDF 104 kb)

## Abbreviations

ANCOVA: Analysis of covariance
ASA: American Society of Anesthesiologists
BMI: Body mass index
eCRF: Electronic case report form
EHS: European Hernia Society
EQ5D-5 L: Euro Quality of Life-5D-5 L
EuraHS-QoL: EuraHS Quality of Life
MCQ: Medical cost questionnaire
MEC-U: Medical Research Ethics Committees United
MMC: Maxima Medical Center
MRI: Magnetic resonance imaging
NRS: Numeric rating scale
NTR: Netherlands Trial Register
OLVG: Onze Lieve Vrouwe Gasthuis
PCQ: Productivity cost questionnaire
QALY: Quality-adjusted life year
RKZ: Rode Kruis Ziekenhuis
TEP: Totally extraperitoneal
